# Blood Samples of Peripheral Venous Catheter or The Usual Way: Do Infusion Fluid Alters the Biochemical Test Results?

**DOI:** 10.5539/gjhs.v8n7p93

**Published:** 2015-11-03

**Authors:** Mahboobeh Taghizadeganzadeh, Mohammadreza Yazdankhahfard, Mohammadreza Farzaneh, Kamran Mirzaei

**Affiliations:** 1 Nursing Graduate Student Bushehr University of Medical Sciences, Bushehr, Iran; 2 Faculty of Nursing and Midwifery, Bushehr University of Medical Sciences, Bushehr, Iran; 3 Department of Pathology and Molecular and Cytogenetic Fellowship, Department of Pathology, University of Bushehr, Bushehr, Iran; 4 Associate Professor of Community Medicine, Bushehr University of Medical Sciences, Bushehr, Iran

**Keywords:** biochemical, peripheral vein catheter, phlebotomy

## Abstract

**Background:**

Most blood tests require venous blood samples. Puncturing the vein also causes pain, infection, or damage to the blood, and lymph flow, or long-term healing. This study aimed to determine and compare the biochemical laboratory value of the blood samples that were provided through: peripheral vein infusion (PVI) receiving continuous intravenous fluid; and the usual method of blood sampling.

**Methods:**

This is an interventional, quasi-experimental, and controlled study. The selected study sample included 60 patients, who were hospitalized during 2014, in the Internal Medicine, part of Martyrs of Persian Gulf, teaching hospital at Bushehr. Three blood samples were taken from each patient that were provided through PVI line (5 ml blood collected at beginning of IVC and then another 5 cc), and another case was prepared by common blood sampling (control). All the samples were analyzed in terms of sodium, potassium, urea and creatinine using SPSS Ver.19 software, by paired *t*-test and Pearson’s correlation coefficients.

**Results:**

There was a statistically significant difference between the amount of sodium and potassium in the first blood samples taken from the intravenous infusion line and vein puncture. However, no significant differences were found among the biochemical amount in the second blood samples taken from the intravenous infusion line and vein puncture.

**Conclusions:**

We can use blood samples taken from peripheral intravenous infusion lines after 5cc discarding from the first part of the sample for measuring the value of sodium, potassium, urea and creatinine.

## 1. Introduction

Laboratory diagnostics is an essential part of the clinical decision making, provided that a high degree of quality is established through- out the total testing process (Plebani & Lippi, 2010). Several lines of evidence attest that the manually intensive activities of the pre analytical phase are more prone to uncertainties and errors than those belonging to the analytical and post-analytical phases (Lippi et al., 2011; Lippi, Guidi, Mattiuzzi, & Plebani, 2006). Many errors in testing process are often related to the human errors in the phases before analysis, including sample collection, and their manipulation at health centers and hospitals (Becan-McBride, 1999; Li. Fang, S. Fang, Chung, & Chien, 2008; Lippi, Mattiuzzi, & Guidi, 2006; Lippi, Avanzini, & Cervellin, 2013; Lippi, Salvagno, Montagnana, Franchini, & Guidi, 2006).

Most blood tests require Venous blood samples collection of diagnostic blood specimens for routine clinical chemistry, immunochemistry, coagulation as well as hematological testing is traditionally performed by the phlebotomist with the aid of the tourniquet (Lippi et al., 2006). Vein puncture is a painful and invasive technique that can cause bruising, hematomas, infections, vasovagal reactions, and in rare cases, peripheral nerve injury (Asheghan, Khatibi, & Holisaz, 2011). An important issue here is the patient’s satisfaction with pain management, which is one of the five results related to nursing interventions and can affect the patient’s decisions about seeking care, changing the current medical programs, and following the prescribed treatment (Beck et al., 2010). Moreover, it also exposes nurses to the risk of an accidental needle stick (Carlson et al., 1990).

It is now nearly four decades that sampling is studied to perform diagnostic tests through the arterial and venous catheters (Zand, Rezaie, & Koohestani, 2010). Studies suggest that blood sampling from an existing peripheral saline lock is best suited to patients undergoing multiple blood sampling necessitated by short term investigative procedures or crisis management (Zand et al., 2010; Corbo, Fu, Silver, Atallah, & Bijur, 2007; Hambleton, Gomez, & Andreu, 2014; Seemann & Reinhardt, 2000).

Additional studies have explored performing vein puncture proximal or distal to a peripheral intravenous infusion (Ong, Boykin, & Barnett, 1979; Read, Viera, & Arkin, 1988). Several studies have been conducted that compare laboratory values of blood drawn via IV catheters after discarded amount of it to laboratory values of blood obtained via vein puncture (Berger-Achituv, Budde-Schwartzman, Ellis, Shenkman, & Erez, 2010; Braniff, DeCarlo, Haskamp, & Broome, 2014; Zengin & Enc, 2008) or have been conducted that Determine Optimal Waste Volume From an Intravenous Catheter to obtain reliable samples from IV catheter (Baker et al., 2013)

But, typically nurses, or laboratory staff use the venous blood by vein puncture, despite the peripheral catheters are available (Zlotowski Steven, Kupas Douglas, & Wood, 2001). Hence, the injection fluid and drugs change results (Ohnishi, 2005).

Yet, only a few studies have compared biochemical parameters in blood samples obtained from vein puncture and peripheral catheter while the patient is receiving intravenous fluids—and most of the few existing studies (Herr Robert, Bossart Philip, Blaylock, Kroger, & Ash, 1990) end with the researchers’ emphasis on the need for more research with more samples, therefore this study was conducted to determine and compare the laboratory results of biochemical value, which are obtained from PVI and conventional venous blood sampling.

### 1.1 Research Aim

Our main aim was to evaluate the equivalence between analytic biochemical parameters from blood samples obtained from a PVI line and those obtained by vein puncture. Our hypothesis was that blood samples extracted from a PVI line did not differ from those extracted by vein puncture. Thus, patients can be protected from the injuries resulting through sampling by vein puncture. Also nurses can be protected from the risk of an accidental needle stick.

## 2. Method

### 2.1 Participants

This is a quasi-experimental, interventional, and controlled study that was performed on the patients, who were hospitalized in the internal wards of Persian Gulf hospital in Bushehr. The number of participants who were sampled was 60 patients that were calculated by using of Altman normogram with consideration of 95% confidence interval; 90% studying power, and 0.8 standard deviation.

### 2.2 Procedure

After explained the objectives and methodology, written consent was obtained from eligible patients for this study.(patients who needed IV fluid therapy, received at least 100 ml fluid through IV line, were able to give consent, had only one peripheral line placed in one upper extremity and aged >18 years old). We excluded patients who had anemia (hemoglobin level<9 g/dl), vascular disease or coagulopathy, who were immunocompromised; who had difficult venous access, and in whom vein punctures had to be minimized; as well as those who refused to participate. Study data sheets were completed, in clouding the subject’s age, gender, type of IV solution, amount of solution infused and information about the laboratory results of blood samples which were drown.

Three blood samples were taken from each patient. Two samples were obtained through the PVI line that was receiving IV fluid, and another sample was obtained by usual method of blood sampling (vein puncture) with 10 ml syringes and 20 gauge needles. Consequently 180 blood samples were taken.

### 2.3 Method of Blood Sampling

Before of blood sampling, both of areas (PVI line and vein puncture sites) were disinfected with povidone iodone 10% by exported nurse, then sites were washed and cleaned with alcohol 70% to ensure that no povidone iodine was left. Afterward PVI was stopped for 30 second and the tourniquet was placed upper PVI site for 30 second, so PVI before sampling was stopped for 1 minute. Then the IV tubing was disconnected from the IV catheter and two 10 ml syringes were used to aspirate two 5-ml blood from the IV catheter. The first sample poured in laboratory tube (A) and second sample poured in laboratory tube (B). Third 5 ml blood sample was taken by vein puncture from other hand after placed tourniquet for 30 second then it was poured in laboratory tube (C). Further, the false names had been assigned for these samples because the researchers did not want the laboratory technician understand the sampling method, and they were immediately sent for analysis to the laboratory. Biochemical tests of urea and creatinine were conducted based on the enzymatic method with Selectra XL model, and the tests on sodium and potassium were conducted based on atomic absorption, with the flame photometer.

### 2.4 Data Analysis

SPSS ver. 19 software was applied to analyze the information of descriptive statistics (frequency and mean), and analytic (pair *t*-test and Pearson correlation coefficient) due to the variable distribution at the 0.05 significant level.

### 2.5 Ethics Statement

This study was approved and authorized by the ethics committee of Bushehr University of Medical Sciences in Iran.

## 3. Result

Sixty volunterees were enrolled in the study that sixty percent of them were males and fourty percent were females, and their age ranged in age from 19 to 91 years (46.95±20.73). The common cause of hospitalization in the patients was cellulitis (10%). About 58.3% of the participants received 1/3, 2/3 serum, while 41.70% of them received normal saline 0.9% serum through the PVI line. The lowest amount of received fluid until the blood sampling was 2,000 L, and the highest amount was 9,000 L (5008.33±1923.65).

When compared the results of biochemical experiments between samples that were obtained from PVI line “A”, and common method of blood sampling “C”(table 1), by paired *t*-test, statistically significant differences were found only between amount of sodium and potassium (*P* < 0/05), whereas there is no statistically significant difference found in between other values. Also a direct and positive correlation was found between the values of both the blood samples (“A” and “C”) by Pearson correlation coefficient. (Na = 0.83, K= 0.86, BUN = 0.84, Cr = 0.71) that the correlation was statistically significant (*P* = 0/00).

**Table 1:** The mean and Standard Deviation of biochemical tests for first blood sample, which was drawn through peripheral vein infusion (PVI) line and conventional method of blood sampling

Sampling method Variable	(Mean ±STD)		T(59)	P value

	first blood sample	The usual method		
Na*	132.58(± 4.66)	133.37(±5.27)	2.08	0.04
K*	4.25(±0.78)	4.52(±0.81)	4.90	0.00
BUN**	13.18(±8.74)	14.68(±11.18)	1.91	0.06
Cr**	1.17(±0.84)	1.32(±1.31)	1.29	0.20

* meq/l;

** mgr/dl.

Compared the results of biochemical experiments between samples were obtained from PVI line “B”, and common method of blood sampling “C” (Table 2), by paired *t*-test showed that there was no statistically significant difference between the Laboratory values of both the blood samples (p value>0/05). Also a direct and positive correlation was found between the values of second blood samples (“B” and “C”) by Pearson correlation coefficient. (Na = 0.88, K = 0.90, BUN= 1.00, creatinine= 1.00) that the correlation was statistically significant (*P* = 0/00).

**Table 2:** The mean and Standard Deviation of biochemical tests for second blood sample, which was drawn through peripheral vein infusion (PVI) line and conventional method of blood sampling

Sampling method Variable	(Mean ±STD)		T(59)	P value

	second blood sample	The usual method		
Na*	133.60(± 4.60)	133.37(±5.27)	-1.14	0.48
K*	4.50(±0.83)	4.52(±0.81)	-1.60	0.65
BUN**	14.57(±10.88)	14.68(±11.18)	-0.15	0.37
Cr**	1.32(±1.27)	1.32(±1.31)	-1.00	0.49

* mEq/l;

** mgr/dl.

Compared the results of biochemical experiments between samples that were obtained through PVI line “A” and “B” (Table-3), by paired *t*-test, showed a statistically difference between amount of sodium and potassium (*P* < 0/05), while there was not statistically significant difference between in other values. Further, a direct and positive correlation was seen between the values of both the blood samples (“A” and “B”) by Pearson correlation coefficient. (Na= 0.80, K = 0.90, BUN = 0.84, Cr = 0.69) that it was statistically significant (*P* = 0/00).

**Table 3:** The mean and Standard Deviation (SD) of biochemical tests for first and second blood samples, which were drawn through peripheral vein infusion (PVI) line

Sampling method Variable	(Mean ±STD)		T(59)	Pvalue

	first blood sample	second blood sample		
Na*	132.58(± 4.66)	133.60(± 4.60)	2.70	0.01
K*	4.25(±0.78)	4.50(±0.83)	5.19	0.00
BUN**	13.18(±8.74)	14.57(±10.88)	1.79	0.08
Cr**	1.17(±0.84)	1.32(±1.27)	1.24	0.22

* mEq/l;

** mgr/dl.

## 4. Discussion

Several studies show that, in addition to the common method of blood sampling (vein puncture), we can place and keep an intravenous catheter to obtain blood samples for diagnostic tests, without any need for invasive techniques (Schallom & Bisch, 2001). Although other studies has been performed about related this issues, such as, determining of hemolysis in blood samples, which were obtained from different methods of blood sampling, or to estimate the amount of discarded blood before sampling (Baker Rachel et al., 2013; Bowen Raffick, Hortin Glen, Csako, Otanez, & Remaley Alan, 2010; Heiligers-Duckers, Peters Nathalie, van Dijck Jose, Hoeijmakers Jan, & Janssen Marcel, 2013; Himberger John & Himberger Laura, 2001; Lippi, Cervellin, & Mattiuzzi, 2013) But, typically nurses, or laboratory staff use the venous blood by vein puncture (Zlotowski Steven et al., 2001), despite the peripheral catheters are available Hence, the injection fluid and drugs change results (Ohnishi, 2005).

The present study shows that there is no significant difference between the amounts of sodium, potassium, urea and creatinine in the second blood samples obtained from the peripheral intravenous infusion lines of patients who were constantly receiving fluids through their intravenous infusion lines on one hand and the amounts of the above-mentioned parameters in samples taken by vein puncture. This finding is in agreement with the results of Berger’s study (that was performed on 47 children in 2004 to examine the possibility of replacing the common method of blood sampling with taking blood samples from peripheral intravenous infusion catheter) (Berger-Achituv, Budde-Schwartzman, Ellis Martin, Shenkman, & Erez, 2010), himberger’s study (that was performed on 46 patients to determine accuracy of results biochemical tests and complete blood count in samples that were taken from peripheral intravenous infusion catheters and by vein puncture in1998)(Himberger john & Himberger laura, 2001), Mohler’s study (that was performed to examine the reliability of the blood samples were taken through peripheral intravenous infusion lines, saline lock and vein puncture from 55 patients for complete blood count and electrolytes in 1998) (Mohler, Sato, Bobick, & Wise, 1998), and Watson’s study (that perfrmed to compare the laboratory results of blood samples that were taken from the peripheral intravenous infusion line after was stopped the infusion fluids for 2 minutes with blood samples were taken from the opposite patient’s arm in 1983)(Watson, O’Kell, & Joyce, 1983).

However, the researches of Zlotowski et al. (to compare several experimental parameters from the analysis of blood samples which were obtained from Vein puncture and Saline Lock after injection of 200 ml of normal saline bolus on 30 patients in Emergency Department) was performed, as our study showed that there were no statistically significant differences between the amount of sodium, urea and creatinine in blood samples taken from the peripheral vein catheter and vein puncture, but in contrast to our study, there were significant differences between the amounts of potassium in the samples (Zlotowski Steven et al., 2001).

The study by Roberts Herr et al. (to determine the validity of blood samples that were aspirated through PVI catheter and vein puncture for testing blood cell counting and biochemistry on 38 patients was performed) concluded that there were significant differences found between potassium and sodium, which contrasts with the results of the our study although, it was agree with our results study about of quantities of urea and creatinine (Herr Robert et al., 1990).

The results of the study also show that there were significant differences among the amounts of sodium and potassium in the first blood samples and second blood samples that were taken from the peripheral intravenous infusion lines of patients who were constantly receiving fluids through their peripheral intravenous infusion lines, and the samples were taken by the common method.

## 5. Conclusions

The results of this study showed few differences with other studies that might be caused by different number of participants, receiving different levels of infusion fluids, and differences between the stopping duration of fluid flow before preparing the blood samples from IVC places. These differences showed that there is a need for further research on this issue. So, according to results of this study we can say that blood sampling through IVC line is reliable method to obtained blood samples of patients hospitaliz for biochemical laboratory testing. Thus, patients can be protected from the injuries resulting through sampling by vein puncture.
